# Designs of trials assessing interventions to improve the peer review process: a vignette-based survey

**DOI:** 10.1186/s12916-018-1167-7

**Published:** 2018-10-15

**Authors:** Amytis Heim, Philippe Ravaud, Gabriel Baron, Isabelle Boutron

**Affiliations:** 1 INSERM, U1153 Epidemiology and Biostatistics Sorbonne Paris Cité Research Center (CRESS), Methods of Therapeutic Evaluation of Chronic Diseases Team (METHODS), Paris, France; 2 Paris Descartes University, Sorbonne Paris Cité, Paris, France; 3 Centre d’Epidémiologie Clinique, Hôpital Hôtel-Dieu, Assistance Publique des Hôpitaux de Paris, Paris, France

**Keywords:** Peer review, Randomized controlled trials, Design, Quality, Validity

## Abstract

**Background:**

We aimed to determine the best study designs for assessing interventions to improve the peer review process according to experts’ opinions. Furthermore, for interventions previously evaluated, we determined whether the study designs actually used were rated as the best study designs.

**Methods:**

Study design: A series of six vignette-based surveys exploring the best study designs for six different interventions (training peer reviewers, adding an expert to the peer review process, use of reporting guidelines checklists, blinding peer reviewers to the results (i.e., results-free peer review), giving incentives to peer reviewers, and post-publication peer review).

Vignette construction: Vignettes were case scenarios of trials assessing interventions aimed at improving the quality of peer review. For each intervention, the vignette included the study type (e.g., randomized controlled trial [RCT]), setting (e.g., single biomedical journal), and type of manuscript assessed (e.g., actual manuscripts received by the journal); each of these three features varied between vignettes.

Participants: Researchers with expertise in peer review or methodology of clinical trials.

Outcome: Participants were proposed two vignettes describing two different study designs to assess the same intervention and had to indicate which study design they preferred on a scale, from − 5 (preference for study A) to 5 (preference for study B), 0 indicating no preference between the suggested designs (primary outcome). Secondary outcomes were trust in the results and feasibility of the designs.

**Results:**

A total of 204 experts assessed 1044 paired comparisons. The preferred study type was RCTs with randomization of manuscripts for four interventions (adding an expert, use of reporting guidelines checklist, results-free peer review, post-publication peer review) and RCTs with randomization of peer reviewers for two interventions (training peer reviewers and using incentives). The preferred setting was mainly several biomedical journals from different publishers, and the preferred type of manuscript was actual manuscripts submitted to journals. However, the most feasible designs were often cluster RCTs and interrupted time series analysis set in a single biomedical journal, with the assessment of a fabricated manuscript. Three interventions were previously assessed: none used the design rated first in preference by experts.

**Conclusion:**

The vignette-based survey allowed us to identify the best study designs for assessing different interventions to improve peer review according to experts’ opinion. There is gap between the preferred study designs and the designs actually used.

**Electronic supplementary material:**

The online version of this article (10.1186/s12916-018-1167-7) contains supplementary material, which is available to authorized users.

## Background

The peer review process is the cornerstone of research [1–3]. This process aims to provide a method for rational, fair, and objective decision-making and to raise the quality of publications. However, this process is increasingly being questioned [4]. Primary functions of peer reviewers are poorly defined, and often expectations of manuscripts differ between editors and peer reviewers [5]. Peer review frequently fails to be objective, rational, and free of prejudice [6]. Flawed and misleading articles are still being published [7]. Less than half of biomedical academics think that the peer review process is fair, scientific, or transparent [8]. Studies have highlighted some limitations of peer review [9–11], including limitations in detecting errors and fraud, improving the completeness of reporting [12], or decreasing the distortion of study results [13].

Some interventions developed and implemented by editors to improve the quality of peer review include blinding the peer reviewer to the author’s identity, using open peer review, or training peer reviewers [14]. However, research evaluating these interventions with an experimental design is scarce [15]. Furthermore, assessing these interventions can raise important methodological issues related to the choice of study type, setting, and type of manuscript being evaluated [15].

Here, we used a vignette-based survey of experts to determine the best study designs for assessing interventions to improve the peer review process according to experts’ opinions. Furthermore, for interventions that were previously evaluated [15], we determined whether the study designs actually used were the study designs experts preferred.

## Methods

### Study design

We performed a series of vignette-based surveys. A vignette can be defined as a hypothetical situation for which research participants are asked a set of directed questions to reveal their values and perceptions. The vignette-based survey has been found useful in different biomedical fields. It is frequently used to examine judgments and decision-making processes and to evaluate clinical practices [16, 17]. The method has also been used to identify the best trial designs for methodological questions [18, 19]. In this study, vignettes were case scenarios of trials assessing different interventions aimed at improving the quality of peer review.

### Vignettes’ conception

To build the vignettes, we performed a methodological review to identify a variety of interventions for improving peer review.

#### Methodological review

We searched MEDLINE (via PubMed), with no restriction on language or date of publication. Our search strategy relied on the search terms “peer review,” “peer reviews,” “peer reviewer,” or “peer reviewers” in the title. We included all types of experimental designs evaluating any intervention aiming to improve the quality of the peer review process in biomedical journals. We also included all articles (including editorials, comments) highlighting an intervention to improve the peer review process. The title and abstract of papers were screened by one researcher (AH) for eligibility.

**Fig. 1:**
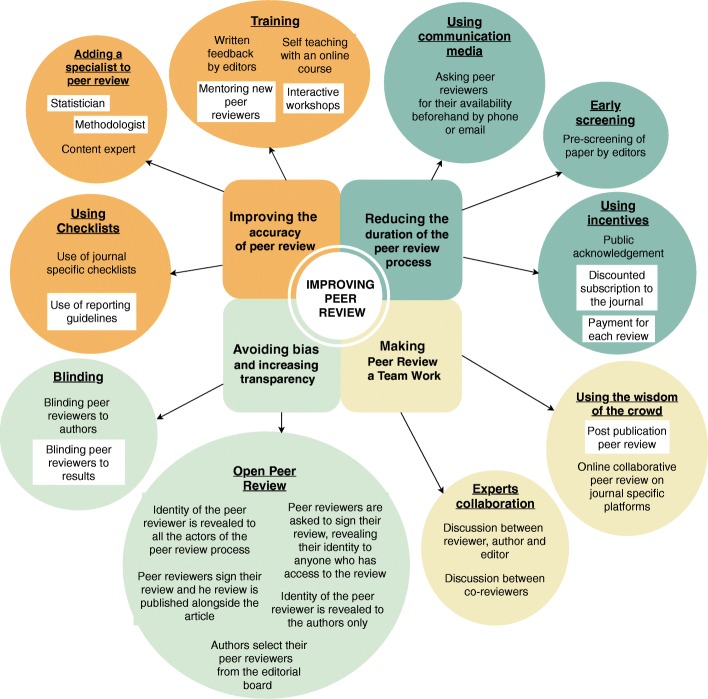
Interventions for peer review identified and classified. Interventions selected to be explored in the vignette-based survey are highlighted in a white box

A total of 12 interventions were identified. Interventions were classified according to their goal (Fig. 1): (1) to improve the accuracy of peer review (i.e., training; adding a specialist to peer review; using checklists); (2) to avoid bias and increase transparency (i.e., blinding; open peer review); (3) to reduce the duration of the peer review process (i.e., using communication media; early screening; use of incentives such as payment), and (4) to make peer review a team effort (i.e., using the wisdom of the crowd such as post publication peer review and expert collaboration).

Six different interventions were selected: training peer reviewers, adding an expert to the peer review process, use of reporting guidelines checklists, blinding peer reviewers to the results (i.e., results-free peer review), giving incentives to peer reviewers, and post-publication peer review. These interventions are described in Table 1.

**Table 1:** Interventions included in the vignette-based survey

Intervention	Description
Training	Peer reviewers are asked to attend an online training program, with lessons on how to evaluate the methodology, the reporting of data and results, the ethical issues, and how to address them in a review. The course will also inform peer reviewers on what journals want from them from an editor’s perspective. Peer reviewers are then supervised for three articles specifically selected for the course.
Adding an expert to the peer review process	An expert is asked to peer review the manuscript in addition to the usual peer review process. The expert should be a statistician or a methodologist.
Use of reporting guidelines checklist	Peer reviewers are asked to complete a checklist based on guidelines (such as CONSORT or STARD, depending on the nature of their manuscript), in addition to their usual review. The checklist is then sent to the authors so they can revise their manuscript.
Results-free peer review	Peer reviewers are blinded to the results of the study. The peer review process unfolds in 2 steps: 1. Peer reviewers receive the manuscript without the abstract, results or discussion. They write a first review and make a recommendation for publication. This first review is sent to the editor. 2. Peer reviewers then receive the full manuscript to comment on the results, discussion and abstract by answering two simple questions on the completeness of the reporting and on the validity of the interpretation. The review is sent to the editor and combined with the first one.
Use of incentives	Reviewers are told they will receive an incentive (payment or discounted subscription to the journal) when they are asked to peer review the manuscript.
Post-publication peer review	Manuscripts are posted online on an open access platform where researchers from all around the world with any background can peer review the study. Chosen researchers are also actively invited by the author and the editor to peer review the online publication. The peer review is entirely transparent: the reviewers’ names and affiliation, their report and the approval status they choose are published along with the article. Peer review reports are posted as soon as they are received and the peer review status of the article is updated with every published report. Once an article has passed peer review (i.e., it has received at least two “Approved” statuses from independent peer reviewers), it will be indexed in PubMed, PubMed Central, Scopus, and Embase.

The choice of these interventions took into account the following factors: having at least one intervention within each group and making sure that the interventions’ assessment raised different methodological issues and consequently required different types of study design. For this purpose, we selected interventions that targeted the peer reviewers (e.g., training, incentives) or the manuscript (e.g., adding a specialist) or involved important changes in the process (e.g., post-publication peer review). Furthermore, we favored interventions that we believed were important in terms of their goal (improving the accuracy of peer review and avoiding bias), were implemented but never tested (blinding peer reviewers to results; post-publication peer review), or were frequently suggested (use of incentives).

More specifically, we decided to consider three interventions aimed at improving the accuracy of peer review (i.e., training, adding a specialist to peer review, using checklists), which we believe is a very important goal of the peer review. The intervention “results-free peer review” was selected because of clear evidence of outcome bias in the peer review process [20], and some editors (e.g., *BMC Psychology*) have implemented this new form of review. Nevertheless, the intervention has never been evaluated. Use of incentives is regularly highlighted as being essential to improve the peer review process, and some initiatives such as Publon are being implemented. Finally, post-publication peer review is widely implemented in some fields and is increasingly been used in biomedical research with specific publishers such as F1000. However, this new process has never been evaluated.

#### Vignettes’ content

**Fig. 2:**
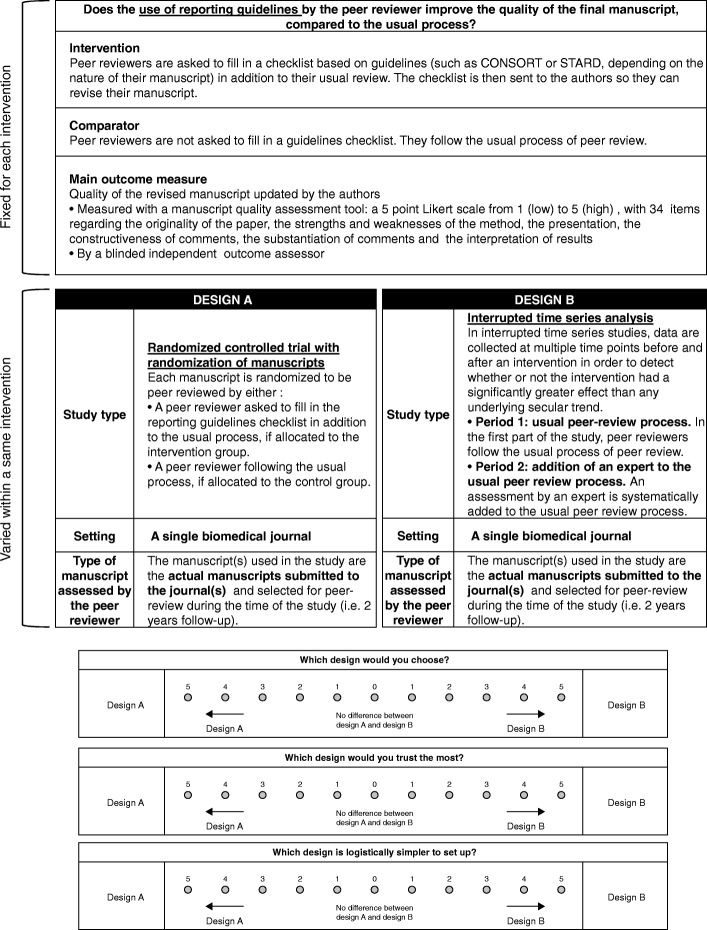
Template of the vignette and survey questions

The vignettes were structured in two parts as shown in Fig. 2. The first part described the study objective. It included the description of the intervention, the comparator (i.e., usual process of peer review), and the main outcome measure (i.e., quality of the peer review report or quality of the manuscript revised by the authors according to the type of intervention assessed) and remained unchanged for all vignettes.

The second part of the vignette described the study design considering three different features: the study type, setting, and type of manuscript assessed by the peer reviewer when appropriate; each of these three features varied among the vignettes (Fig. 2). The study type could be an RCT randomizing manuscripts, an RCT randomizing peer reviewers, a cluster RCT randomizing journals, an interrupted time series analysis, a pairwise comparison, or a stepped wedge cluster RCT with randomization of journals (Table 2). The setting could be a single biomedical journal, several biomedical journals from a single publisher, or several biomedical journals from several publishers. The type of manuscript assessed by the peer reviewer could be the actual manuscripts received by the journal(s) or a fabricated manuscript that purposely included methodological issues, errors, and poorly reported items.

**Table 2:** Study types

Study type	
RCT with randomization of manuscripts	Each manuscript is randomized to be peer reviewed by a peer reviewer in the intervention group or a peer reviewer in the control group.
RCT with randomization of peer reviewers	Peer reviewers are randomized to the intervention group or the control group.
Cluster RCT with randomization of journals	Journals are randomized to the intervention group or the control group. All peer reviewers from a journal follow the same peer review process.
Interrupted time series analysis	Data are collected at multiple time points before and after an intervention to detect whether the intervention had a significantly greater effect than any underlying secular trend. • Period 1: Peer reviewers follow the usual process of peer review. • Intervention: All peer reviewers follow the process of the intervention. • Period 2: Manuscripts are evaluated after the peer review process.
Pairwise comparison	Each manuscript is sent to be reviewed by both a peer reviewer from the intervention group AND a peer reviewer from the control group.
Stepped wedge cluster RCT with randomization of journals	The intervention is rolled out sequentially to the journals over a number of time periods. • During the first period, none of the journals follow the intervention. • During the second period, one journal is randomized to invite its peer reviewers to participate in the intervention peer review process. The other journals continue the usual process. • During the third period, an extra journal is randomized to participate in the intervention peer review process, and the other journals continue with the usual process. Therefore, two journals are undergoing the intervention during this period. This process of randomization is repeated at each period until the last journal finally joins the intervention group.

All possible combinations of designs were generated, and two methodologists assessed each design to exclude implausible and contradictory ones. Particularly, we considered a single type of manuscript (i.e., actual manuscripts received by the journal[s]) for the following interventions: adding an expert to the peer review process, use of reporting guidelines checklists, use of incentives, and post-publication peer review.

### Participants

Our target population consisted of researchers with an expertise in the field of peer review or methodology of clinical trials. To recruit such participants, we searched for the email addresses for all the authors of the papers of our review. We also identified and searched for the email addresses of participants of the 2013 Peer Review Congress, members of the Editorial Boards of the five journals with the highest impact factor, the Journal of Clinical Epidemiology and Public Library of Science Medicine (PLOS); members of the Enhancing the QUAlity and Transparency Of health Research (EQUATOR) network, the REduce research Waste and Reward DIligance (REWARD) Alliance, the METHODS Cochrane Group, the Methods in Research on Research (MiRoR) project, Trial Forge and Meta-Research Innovation Center at Stanford (METRICS). The full list is available in Additional file 1: Appendix 1.

### Surveys

A total of 94 vignettes were included in the study: 24 for training, 24 for results-free peer review, 13 for the use of reporting guidelines checklist, 10 for adding an expert to the process, 13 for the use of incentives, and 10 for post-publication peer review. Participants received an invitation via email with a personalized link to the survey. On the home page of the website, participants were informed that the data collected was anonymous and were asked to give their informed consent before starting the questionnaire. A maximum of three reminders were sent to participants, and no incentive was used to maximize the response rate. Participants were proposed two vignettes describing two different study designs to assess a same intervention, and had to indicate which study design they preferred (Fig. 2). Each participant was invited to evaluate six pairs of vignettes for a given intervention.

### Sample size

From a pragmatic point of view, we wanted each pair of vignettes to be assessed by participants at least once. For the interventions with fewer than 20 vignettes, we planned for each pair of vignettes to be assessed twice, to increase the number of evaluations per vignette. Therefore, to assess all pairs of vignettes (*n* = 1044 in total: 276 each for training and results-free peer review, 156 each for the use of reporting guidelines checklist and the use of incentives, and 90 each for adding an expert to the process and for post-publication peer review), and assuming each participant would assess six pairs of vignettes, we needed a minimum of 174 participants. If participants could not evaluate six pairs of vignettes, other participants were recruited.

### Ranking of the study designs actually implemented

Using the results of our methodological review, we determined how the study designs actually used were ranked in our survey. For this purpose, we extracted the study type, setting, and type of manuscript used to assess these interventions in the review.

### Outcomes

Our main outcome was the overall preference for a study design. Participants had to answer the following question: “If you had to conduct this trial, which study would you choose?” on a semantic differential scale rated from − 5 (preference for study A) to 5 (preference for study B), 0 indicating no preference between the suggested designs.

Other outcomes were the rankings for trust in the results and feasibility, measured by using the same scale. The questions asked were as follows:

- “If you read the results of this study, which study would you trust most?”
- “Which protocol is logistically simpler to set up?”

Participants had the opportunity to leave comments if they wished to.

### Statistical analysis

Answers for the online questionnaire were collected through the website. The results were recorded in a .csv file and analyzed with R v3.2.2 (http://www.R-project.org, the R Foundation for Statistical Computing, Vienna, Austria) and SAS 9.4 (SAS Institute Inc., Cary, NC). For each intervention and each outcome (overall preference, trust in results, and feasibility), the mean score for each vignette was calculated for each combination of designs in order to have a ranking. For each intervention, we used a linear mixed model to assess the association between each outcome and the following three fixed effects: study type, setting, and type of manuscript. The reading order of the two vignettes of a pair was added as a fourth fixed effect. To account for correlation between vignettes, an intercept term that randomly varied at the level of the vignette effect was included in the model. To account for correlation within vignette pairs (at each comparison, two vignettes have exactly opposite scores), we bootstrapped pairs with 1000 replications of the original sample to estimate the parameters (and 95% confidence intervals) of the model. Correlation due do respondents was found to be null, so it was not modeled.

## Results

### Participants

Between May 11, 2017, and July 31, 2017, 1037 people were contacted in waves until all pairs of vignettes were evaluated. Of the 331 participants who clicked on the link, 210 gave their consent, and 204 completed the survey (Table 3). Participants were located mainly in Europe (*n* = 114, 56%) and North America (*n* = 72, 35%). More than half worked as a methodologist (*n* = 135, 66%) and about half were trialists (*n* = 99, 49%) or editors (*n* = 102, 50%).

**Table 3:** Baseline demographics and other characteristics of participants (*n* = 204)

	No. of participants (%)
Age, years	
< 40	65 (32)
40–50	52 (25)
51–60	45 (22)
60	42 (21)
Sex	
Male	117 (57)
Female	87 (43)
Location	
Europe	114 (56)
North America	72 (35)
South America	0 (0)
Asia	4 (2)
Africa	2 (1)
Oceania	12 (6)
Occupation*	
Methodologist	135 (66)
Trialist	99 (49)
Editor	102 (50)
Other	22 (11)

*Many participants had combined occupations (methodologist and/or trialist and/or editor)

### Vignette-based surveys

Additional file 1: Appendix 2 summarizes the results in a spider diagram of mean vignette scores per intervention in terms of overall preference, trust in the results, and feasibility.

#### Preferred study designs

Additional file 1: Appendix 3 provides the mean score for each vignette for each combination of features (i.e., study type, setting, manuscript type). Table 4 reports the factors associated with overall preference for each study design feature (study type, setting, type of manuscript). For each feature, we arbitrarily identified a reference (*stepped wedge cluster RCT with randomization of journals* for the study type, *several biomedical journals from different publishers* for the setting, and *one fabricated manuscript* for the type of manuscript. The parameter reported is the mean difference in overall preference associated with each category of independent variable as compared with the reference (after adjusting for all other variables).

**Table 4:** Results—factors associated with overall preference for each study design feature: parameter estimates [and 95% confidence intervals]. For each independent variable, parameter estimates represent mean difference in overall preference associated with each category of independent variable as compared with the reference (after adjusting for all other variables in the table and after taking into account the reading order of the 2 vignettes of the pair)

	Training peer reviewers (24 vignettes, 276 pairs)	Adding an expert to the peer review process (10 vignettes, 90 pairs*)	Use of reporting guidelines checklist (13 vignettes, 156 pairs*)	Results free peer review (24 vignettes, 276 pairs)	Using incentives (13 vignettes, 156 pairs*)	Post-publication peer review (10 vignettes, 90 pairs*)
	Estimate [95% CI]	Estimate [95% CI]	Estimate [95% CI]	Estimate [95% CI]	Estimate [95% CI]	Estimate [95% CI]
Study type						
RCT with randomization of manuscripts	0.92 [-0.50 ; 2.41]	**2.03** **[0.51 ; 3.49]**	**2.69** **[1.39 ; 3.95]**	**2.53** **[1.27 ; 3.76]**	1.00 [-0.21 ; 2.16]	**2.55** **[1.13 ; 4.09]**
RCT with randomization of peer reviewers	**1.45** **[0.14 ; 2.78]**	N/A	1.99 [0.69 ; 3.37]	2.24 [0.98 ; 3.50]	**2.25** **[0.94 ; 3.49]**	N/A
Cluster RCT with randomization of journals	0.30 [-1.12 ; 1.63]	0.76 [-0.90 ; 2.43]	0.34 [-1.25 ; 1.93]	0.63 [-0.56 ; 1.88]	-0.16 [-1.49 ; 1.18]	1.73 [0.13 ; 3.51]
Interrupted time series analysis	-0.10 [-1.48 ; 1.38]	-0.19 [-1.74; 1.39]	0.10 [-1.21 ; 1.44]	0.07 [-1.28 ; 1.40]	0.73 [-0.51 ; 2.02]	1.58 [0.13; 3.15]
Pairwise comparison	0.83 [-0.49 ; 2.18]	N/A	N/A	1.61 [0.35 ; 2.86]	N/A	N/A
Stepped wedge cluster RCT with randomization of journals***	0.00 [-]	0.00 [-]	0.00 [-]	0.00 [-]	0.00 [-]	0.00 [-]
Setting						
Single biomedical journal	-1.02 [-1.82 ; -0.27]	-2.62 [-4.23 ; -0.80]	-2.50 [-3.51 ; 1.50]	-0.20 [-1.03 ; 0.62]	-1.51 [-2.59 ; -0.43]	-3.13 [-4.09 ; -1.53]
Several biomedical journals from a single publisher	-0.21 [-0.84 ; 0.44]	-1.10 [-2.32 ; 0.06]	-0.12 [-1.06 ; 0.80]	**0.18** **[-0.52 ; 0.83]**	-0.01 [-0.82 ; 0.83]	-0.92 [-1.95 ; 0.22]
Several biomedical journals from different publishers***	**0.00** **[-]**	**0.00** **[-]**	**0.00** **[-]**	0.00 [-]	**0.00** **[-]**	**0.00** **[-]**
Type of manuscript						
Actual manuscripts submitted to the journal(s)	**1.04** **[0.37 ; 1.79]**	N/A	N/A	**0.57** **[-0.15 ; 1.26]**	N/A	N/A
One fabricated manuscript***	0.00 [-]	N/A	N/A	0.00 [-]	N/A	N/A

*N/A*, not applicable

Results in bold indicate the most preferred study design features for each intervention

*Indicates the pairs of vignettes for these interventions were assessed twice each

***Indicates reference category for each independent variable

Overall, the preferred study type was RCTs with randomization of manuscripts for four interventions (adding an expert, use of reporting guidelines checklist, results-free peer review, post-publication peer review) and RCTs with randomization of peer reviewers for two interventions (training peer reviewers and using incentives), with adjustment for all other variables. The preferred setting was mainly several biomedical journals from different publishers, and the preferred type of manuscript was actual manuscripts submitted to journals.

Other designs, such as the cluster stepped wedge of journals or the interrupted time series, scored low.

#### Trust and feasibility

Additional file 1: Appendices 4 and 5 provide the mean score for each vignette for each combination of features (i.e., study type, setting, manuscript type) for trust and feasibility. After adjustment for all other variables, the most trusted study designs were consistent with the preferred study designs for all interventions (Additional file 1: Appendix 6). In contrast, the study designs rated first in terms of feasibility were not the preferred study designs (Additional file 1: Appendix 7). The preferred study types in terms of feasibility were a pairwise comparison for training peer reviewers (rated as third preferred study type), a cluster RCT with randomization of journals for results-free peer review and use of reporting guidelines checklists (rated fourth and third preferred study type, respectively), and interrupted time series analysis for adding an expert to the peer review process, using incentives and post-publication peer review (rated last, third and third preferred study types, respectively). The setting and type of manuscript were mainly a single biomedical journal and use of a fabricated manuscript.

#### Ranking of the study designs actually implemented

The ranking of the study design actually implemented is reported in Table 5. Our review identified no studies assessing results-free peer review, use of incentives, and post-publication peer review; five RCTs and one cross-sectional study assessing training; two RCTs assessing use of reporting guidelines checklists and two RCTs assessing adding an expert. None used the designs rated first by experts in terms of preference. None were ranked in the first quarter. This ranking is mainly related to the choice of setting.

**Table 5:** Ranking of the study designs of the RCTs identified in the methodological review of interventions to improve the peer review process according experts

	Studies identified				Ranking according to experts*		
	No. of studies	Study type	Setting	Type of manuscript	Preference	Trust	Feasibility
Training	6	- 5 randomized controlled trial of peer reviewers - 1 cross-sectional study	Single journal	- Real manuscripts - 1 RCT with fabricated manuscript	8/24 (4 RCTs) 21/24 (1 RCT)	11/24 (4 RCTs) 22/24 (1 RCT)	9/24 (4 RCTs) 6/24 (1 RCT)
Use of reporting guidelines checklist	2	Randomized controlled trial of manuscripts	Single journal	Real manuscripts	5/13	5/13	2/13
Adding an expert	2	Randomized controlled trial of manuscripts	Single journal	Real manuscripts	8/10	8/10	1/10

*The cross-sectional design was not included in the vignette study

## Discussion

The peer review process is central to the publication of scientific articles. Our series of vignette-based surveys attempted to surpass the methodological problems of performing research on research by assembling a panel of experts on this research question and using their collective wisdom to identify the best designs. We created 94 vignettes of different study designs for 6 different interventions. Overall, 204 experts in peer review or methodology of clinical trials assessed 1044 paired comparisons of designs, which allowed participants to select their answers in terms of overall preference, trust in the results, and feasibility of the study. We identified the study design that was preferred by experts. We did not specify what is considered the “best” study design because we wanted to give full freedom to the experts and let them balance the different features of the design in terms of internal validity, external validity, and feasibility.

Our study has important strengths. We performed a methodological review to identify interventions for evaluating peer review and to classify them according to their effect on the peer review process. Participants, with expertise as a methodologist, an editor, a trialist or involved in research on peer review, were well suited to compare and score the vignettes. The vignette-based survey we used is an innovative study design [18], which, to our knowledge, has never been used in the context of peer review. This method also allowed experts to discuss the pros and cons of each designs. Table 6 provides the notable characteristics of the preferred study designs for each intervention.

**Table 6:** Notable characteristics of the preferred designs for each intervention

Intervention	Comments on the best study design according to experts
Training intervention	The design recommended by the experts was an RCT with randomization of peer reviewers, set in several biomedical journals from different publishers, using actual manuscripts submitted to the journal. The choice of an RCT with randomization of peer reviewers has the advantage of being close to the real-life procedures of the peer review process, with the benefit of using randomization. The issue with the training intervention is its length in time. This raises issues related to poor adherence and missing outcome when peer reviewers randomized never assess a manuscript. The pairwise comparison was the second-ranked design. This design has the advantage of addressing the issue of manuscript variability, thus increasing statistical power, and avoiding the loss to follow-up problem, because no long-term follow up is needed. Such design has never been used to our knowledge. The cluster RCT and stepped wedge cluster RCT were not often chosen by the participants because of the risk of contamination, because peers can review for more than one journal at a time.
Addition of an expert (methodologist or statistician)	The addition of an expert to the peer review process was preferably assessed with an RCT of manuscripts, set in several journals from different publishers, using the actual manuscripts submitted to the journal. The cluster RCT was the second preferred design for all three of the outcomes. This design has the advantage of including a large variety of reviewers and manuscripts, and it is logistically easy for the editors who do not have to change process for each manuscript. It is nevertheless a difficult design to put in place, as shown by its systematically low score in the feasibility rankings, and a very large number of clusters would be needed to compensate for the high variability between journals (publisher, editorial policies, subject area, quality of reviewers etc.). The interrupted time series set in a single journal was the preferred design in terms of feasibility. This study type is not randomized, which could potentially create bias.
Use of reporting guidelines checklist	The favored designs to assess the use of reporting guidelines checklist was an RCT of manuscripts, set in several biomedical journals from several or a single publisher, using actual manuscripts. The choice to randomize manuscripts rather than peer reviewers is interesting in terms of logistics, because manuscripts receiving the intervention can be sent directly with the checklist. The preferred settings give a good external validity to the study.
Results-free peer review	Our analysis suggests the factor influencing the most participant’s decision in their overall preference was the type of study. The favorite type of studies overall were the RCT of peer reviewers and the RCT of manuscripts. The choice of an RCT randomizing manuscripts for the results-free peer review seems appropriate because the intervention is held directly on the manuscript. The issue with the randomization of manuscripts in this situation is the possibility for peer reviewers to perform both with-results and results-free reviews. With the intervention having a potential learning effect, it would artificially increase the quality of reviews in the control group. This intervention has—to our knowledge—never been assessed, which is notable as it could help reduce the important bias towards positive results.
Use of incentives	The use of incentives raised interesting comments from participants. Particularly, they highlighted that the existence of an incentive may encourage reviewers to accept invitations even if not fully qualified. In a similar way, reviewers in the incentive arm are likely to accept more reviews than the control arm, which raises some issues for the design.
Post-publication peer review	It was one of the most innovative intervention we included in our study. Although this system has already been in place in several journals, such as F1000, it has, to our knowledge, never been assessed. This intervention is interesting because it changes the entire peer review process, not just the way peer reviews assess the manuscripts. The preferred type of study for this intervention was the randomization of manuscripts. Being randomized, this design would indeed lower the risk of bias of the study; however, it may be hard to implement such an intervention, because journals would have to manage two completely different peer review systems at the same time.

Our results revealed that the preferred designs were often very similar to the most trusted designs but very different from the most feasible ones. Preferred settings were generally in several biomedical journals from one or more publishers, and the preferred type of manuscript assessed by the peer reviewer was always an actual manuscript submitted to the journal. In contrast, the most feasible designs were often set in a single biomedical journal, with assessment of a fabricated manuscript. Some designs, such as RCTs with randomization of manuscripts or peer reviewers, were usually high-ranked. Other designs, such as the cluster stepped wedge of journals or the interrupted time series, regularly scored low.

This preference for trust in the results of the study rather than feasibility could be explained by the fact that the most trusted study designs does not raise important feasibility issues and should be easy to implement. Indeed, there are no major barriers to the randomization of manuscripts or peer reviewer. Opt-out consent procedures and blinding procedures are usually easy to implement; authors and reviewers are informed that studies of peer review are being conducted within a journal but are not informed of the studies to avoid any change in behavior. Outcome assessment (quality of the peer review report or quality of the manuscript) can be assessed by blinded outcome assessors. However, the ability to coordinate between journals and publishers and achieve a required sample size could be considered a major barrier.

Our results also highlighted that the designs actually implemented was never the preferred study design. Particularly, all studies performed involved a single journal, whereas the preferred study designs were set in several medical journals from different publishers, which provides high external validity because it is close to the real-world situation, including many types of journals, manuscripts, and reviewers. This inconsistency between implemented studies and preferred study designs may be due to these trials being the first performed in this field and that investigators, who were pioneers in these fields, favored ensuring feasibility. Furthermore, investigators and researchers in this field must have learned a lot from these trials and would probably improve the design of future trials taking into account these previous experiences.

The following limitations should be acknowledged. We focused on 6 interventions of the 12 identified and on the assessment of a single intervention per study, even though the synergistic use of interventions could improve the quality of peer review. Because of the restrictive format of the vignettes, not all elements of study designs could be addressed. No indication of the sample size was included, which could have an effect on both feasibility and trust in the results. The number of vignettes we included in the questionnaire was also limited, which restricted the number of interventions, comparators, and outcomes. Our study focused solely on the interventions improving the quality of peer review and thus of manuscripts, but other innovations such as re-review opt out and portable or cascade peer review were not included [21]. Participation level was about 20%, which could have biased our results. However, the level of expertise of participants was appropriate. Finally, we cannot exclude that participants could have been influenced by ideological or other preferences for a study design for a given intervention.

## Conclusion

Well-performed trials are needed to assess interventions proposed to improve the peer review process. We encourage editors and other investigators to pursue the research on peer review and plan their studies in light of the findings of this vignette-based survey. We hope the evaluation of study designs with a vignette-based survey, based on international expertise, will help to develop a standardization of practices. This standardization will help improve the comparison and ensure the quality of future studies.

## Additional files

Additional file 1: Appendix 1. List of participants. Appendix 2. Results—Spider diagrams of mean vignette scores per intervention in terms of overall preference, trust in the results and feasibility. Appendix 3. Results—Mean score for each combination of features for the preferred study design (primary outcome). Appendix 4. Results—Mean score for each combination of features for trust in results (secondary outcomes). Appendix 5. Results—Mean score for each combination of features for feasibility (secondary outcomes). Appendix 6. Results—Parameter estimates for trust in the results model. Appendix 7. Results—Parameter estimates for feasibility model. (DOCX 1461 kb)

## Abbreviations

95% CI: 95% confidence intervals
RCT: Randomized controlled trials

## References

[1] Smith R. (1997). Peer review: reform or revolution?. BMJ.

[2] Rennie D (1992). Suspended judgment. Editorial peer review: let us put it on trial. Control Clin Trials.

[3] Kronick DA (1990). Peer review in 18th-century scientific journalism. JAMA.

[4] Jefferson T, Rudin M, Brodney Folse S, Davidoff F (2007). Editorial peer review for improving the quality of reports of biomedical studies. Cochrane Database Syst Rev.

[5] Chauvin A, Ravaud P, Baron G, Barnes C, Boutron I (2015). The most important tasks for peer reviewers evaluating a randomized controlled trial are not congruent with the tasks most often requested by journal editors. BMC Med.

[6] Mahoney MJ (1977). Publication prejudices: an experimental study of confirmatory bias in the peer review system. Cogn Ther Res.

[7] The Editors of The L (2010). Retraction—Ileal-lymphoid-nodular hyperplasia, non-specific colitis, and pervasive developmental disorder in children. Lancet.

[8] Ho RC, Mak KK, Tao R, Lu Y, Day JR, Pan F (2013). Views on the peer review system of biomedical journals: an online survey of academics from high-ranking universities. BMC Med Res Methodol.

[9] Wager E, Jefferson T (2001). Shortcomings of peer review in biomedical journals. Learned Publishing.

[10] Rennie D (1999). Misconduct and journal peer review.

[11] Henderson M. (2010). Problems with peer review. BMJ.

[12] Hopewell S, Collins GS, Boutron I, Yu LM, Cook J, Shanyinde M, Wharton R, Shamseer L, Altman DG (2014). Impact of peer review on reports of randomised trials published in open peer review journals: retrospective before and after study. BMJ.

[13] Lazarus C, Haneef R, Ravaud P, Boutron I (2015). Classification and prevalence of spin in abstracts of non-randomized studies evaluating an intervention. BMC Med Res Methodol.

[14] Galipeau J, Moher D, Skidmore B, Campbell C, Hendry P, Cameron DW, Hebert PC, Palepu A (2013). Systematic review of the effectiveness of training programs in writing for scholarly publication, journal editing, and manuscript peer review (protocol). Syst Rev.

[15] Bruce R, Chauvin A, Trinquart L, Ravaud P, Boutron I (2016). Impact of interventions to improve the quality of peer review of biomedical journals: a systematic review and meta-analysis. BMC Med.

[16] Hughes R, Huby M (2002). The application of vignettes in social and nursing research. J Adv Nurs.

[17] Bachmann LM, Mühleisen A, Bock A, ter Riet G, Held U, Kessels AG (2008). Vignette studies of medical choice and judgement to study caregivers’ medical decision behaviour: systematic review. BMC Med Res Methodol.

[18] Do-Pham G, Le Cleach L, Giraudeau B, Maruani A, Chosidow O, Ravaud P (2014). Designing randomized-controlled trials to improve head-louse treatment: systematic review using a vignette-based method. J Invest Dermatol.

[19] Gould D (1996). Using vignettes to collect data for nursing research studies: how valid are the findings?. J Clin Nurs.

[20] Emerson GB, Warme WJ, Wolf FM, Heckman JD, Brand RA, Leopold SS (2010). Testing for the presence of positive-outcome bias in peer review: a randomized controlled trial. Arch Intern Med.

[21] Kovanis Michail, Trinquart Ludovic, Ravaud Philippe, Porcher Raphaël (2017). Evaluating alternative systems of peer review: a large-scale agent-based modelling approach to scientific publication. Scientometrics.

