# Supplementary material for: Designs of trials assessing interventions to improve the peer review process: a vignette-based survey
Source: BMC Med. 2018 Oct 15;16:191. doi: 10.1186/s12916-018-1167-7 (PMC6192007; doi:10.1186/s12916-018-1167-7)
Supplement: Supplementary file 1 — Appendix 1. List of participants. Appendix 2. Results—Spider diagrams of mean vignette scores per intervention in terms of overall preference, trust in the results and feasibility. Appendix 3. Results—Mean score for each combination of features for the preferred study design (primary outcome). Appendix 4. Results—Mean score for each combination of features for trust in results (secondary outcomes). Appendix 5. Results—Mean score for each combination of features for feasibility (secondary outcomes). Appendix 6. Results—Parameter estimates for trust in the results model. Appendix 7. Results—Parameter estimates for feasibility model. (DOCX 1461 kb) [file 12916_2018_1167_MOESM1_ESM.docx]

**APPENDIX**

**Appendix 1** – List of participants

**Appendix 2** – Results – Spider diagrams of mean vignette scores per intervention in terms of overall preference, trust in the results and feasibility

**Appendix 3** – Results – Mean score for each combination of features for the preferred study design (primary outcome)

**Appendix 4** – Results – Mean score for each combination of features for trust in results (secondary outcomes)

**Appendix 5** – Results – Mean score for each combination of features for feasibility (secondary outcomes)

**Appendix 6** – Results – Parameter estimates for trust in the results model

**Appendix 7** – Results – Parameter estimates for feasibility model

**Appendix 1 –** Affiliation of Participants

1. Authors of the articles from the systematic review
2. Corresponding authors of randomized controlled trials published in the Journal of Clinical Epidemiology from 2015 to 2017
3. Editorial board of the Journal of Clinical Epidemiology
4. Members of the International Clinical Trial Methodology Conference
5. Members of the Society for Clinical Trials
6. Members of the Peer Review Congress
7. Corresponding authors of articles on peer review (PubMed search “peer review*”)
8. Members of the Enhancing the QUAlity and Transparency Of health Research (EQUATOR) network
9. Corresponding authors of randomized controlled trials published in Trials from 2016 to 2017
10. Members of the Lancet REduce research WAste and Reward Diligence (REWARD) Statement
11. Members of The Meta-Research Innovation Center at Stanford (METRICS)
12. Staff of the Johns Hopkins Center for Clinical Trials and Evidence Synthesis
13. Members of the METHODS Cochrane Group
14. Members of the EQUATOR/REWARD Conference
15. Professors of the Methods in Research on Research (MiRoR) Project
16. Trial Forge Collaborators
17. Members of the Editorial Boards of the *Journal of American Medical Association*, *British Medical Journal*, *New England Journal of* *Medicine*, *Annals of Internal Medicine*, *PLOS Med* and *the Lancet*

**Appendix 2** – Spider diagrams of mean vignette scores per intervention in terms of overall preference, trust in the results and feasibility

| 1 | RCT of manuscripts / Different publishers / Real manuscripts |
| --- | --- |
| 2 | RCT of manuscripts / Single publisher / Real manuscripts |
| 3 | RCT of manuscripts / One journal / Real manuscripts |
| 4 | RCT of peer reviewers / Different publishers / Real manuscripts |
| 5 | RCT of peer reviewers / Single publisher / Real manuscripts |
| 6 | RCT of peer reviewers / One journal / Real manuscripts |
| 7 | RCT of peer reviewers / Different publishers / Fabricated manuscript |
| 8 | RCT of peer reviewers / Single publisher / Fabricated manuscript |
| 9 | RCT of peer reviewers / One journal / Fabricated manuscript |
| 10 | Pairwise comparison / Different publishers / Real manuscripts |
| 11 | Pairwise comparison / Single publisher / Real manuscripts |
| 12 | Pairwise comparison / One journal / Real manuscripts |
| 13 | Pairwise comparison / Different publishers / Fabricated manuscript |
| 14 | Pairwise comparison / Single publisher / Fabricated manuscript |
| 15 | Pairwise comparison / One journal / Fabricated manuscript |
| 16 | Cluster RCT of journals / Different publishers / Real manuscripts |
| 17 | Cluster RCT of journals / Single publisher / Real manuscripts |
| 18 | Cluster RCT of journals / Different publishers / Fabricated manuscript |
| 19 | Cluster RCT of journals / Single publisher / Fabricated manuscript |
| 20 | Interrupted time series analysis / Different publishers / Real manuscripts |
| 21 | Interrupted time series analysis / Single publisher / Real manuscripts |
| 22 | Interrupted time series analysis / One journal / Real manuscripts |
| 23 | Stepped wedge cluster RCT of journals / Different publishers / Real manuscripts |
| 24 | Stepped wedge cluster RCT of journals / Single publisher / Real manuscripts |


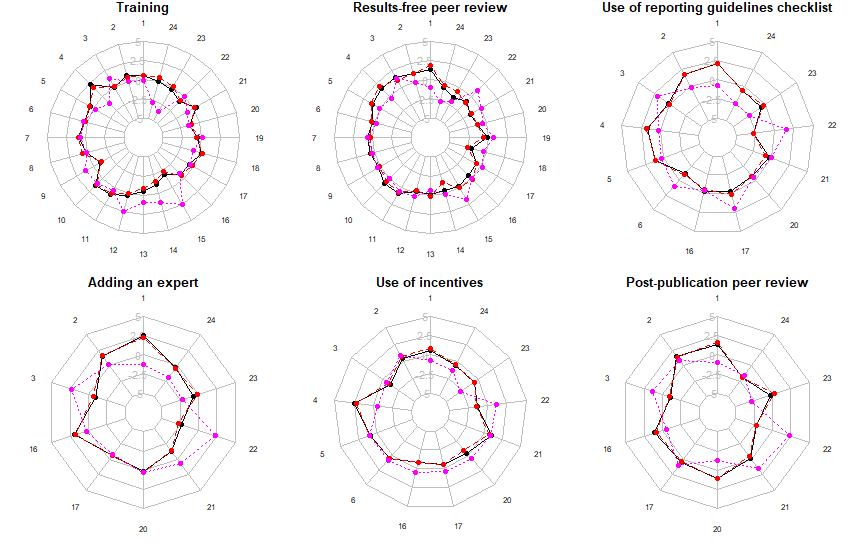


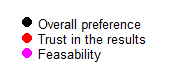


**Appendix 3** – Results – Mean score for each combination of features for the preferred study design

| Study design | | | Interventions | | | | | |
| --- | --- | --- | --- | --- | --- | --- | --- | --- |
| Study Type | Setting | Type of manuscript | TRAINING PEER REVIEWERS  (24 vignettes, 276 pairs)  Mean score (SD)  Rank | RESULTS-FREE PEER REVIEW  (24 vignettes,  276 pairs)  Mean score (SD)  Rank | USE OF REPORTING GUIDELINES CHECKLIST  (13 vignettes, 156 pairs*)  Mean score (SD)  Rank | ADDING AN EXPERT TO THE PEER REVIEW PROCESS  (10 vignettes,  90 pairs*)  Mean score (SD)  Rank | USING INCENTIVES  (13 vignettes, 156 pairs*)  Mean score (SD)  Rank | POST- PUBLICATION PEER REVIEW  (10 vignettes,  90 pairs*)  Mean score (SD)  Rank |
| RCT with randomization of manuscripts | Several biomedical journals from different publishers | **Actual manuscripts submitted to journal(s)** | 0.6 (3.5)  6/24 | 1.3 (3.3)  3/24 | **2.1 (2.4)**  **1/13** | **2.5 (3.0)**  **1/10** | 0.5 (3.2)  4/13 | 1.3 (3.5)  2/10 |
| RCT with randomization of manuscripts | Several biomedical journals from a single publisher | **Actual manuscripts submitted to journal(s)** | 0.8 (3.3)  5/24 | - 1. (3.6)   5/24 | 1.8 (3.1)  2/13 | 1.4 (3.5)  3/10 | 0.5 (3.6)  4/13 | **1.6 (3.1)**  **1/10** |
| RCT with randomization of manuscripts | Single biomedical journal | **Actual manuscripts submitted to journal(s)** | 1. (3.7)   14/24 | **1.5 (3.5)**  **1/24** | 0.3 (3.4)  5/13 | -1.0 (3.7)  8/10 | -1.2 (3.1)  12/13 | -1.1 (3.8)  8/10 |
| RCT with randomization of peer reviewers | Several biomedical journals from different publishers | **Actual manuscripts submitted to journal(s)** | **2.2 (2.8)**  **1/24** | 1.4 (3.4)  2/24 | 1.7 (3.6)  3/13 |  | **2.4 (2.9)**  **1/13** |  |
| RCT with randomization of peer reviewers | Several biomedical journals from a single publisher | **Actual manuscripts submitted to journal(s)** | 0.5 (3.4)  7/24 | 1.2 (3.3)  4/24 | - 1. (3.6)   4/13 |  | 0.9 (3.1)  2/13 |  |
| RCT with randomization of peer reviewers | Single biomedical journal | **Actual manuscripts submitted to journal(s)** | 0.4 (3.9)  8/24 | 0.3 (3.5)  10/24 | -1.3 (4.0)  12/13 |  | 0.5 (4.1)  4/13 |  |
| RCT with randomization of peer reviewers | Several biomedical journals from different publishers | One fabricated manuscript | 1.0 (3.2)  3/24 | 0.7 (3.3)  8/24 |  |  |  |  |
| RCT with randomization of peer reviewers | Several biomedical journals from a single publisher | One fabricated manuscript | 0.2 (3.8)  12/24 | 0.5 (3.5)  9/24 |  |  |  |  |
| RCT with randomization of peer reviewers | Single biomedical journal | One fabricated manuscript | -1.1 (3.6)  21/24 | 0.3 (3.5)  10/24 |  |  |  |  |
| Pairwise comparison | Several biomedical journals from different publishers | **Actual manuscripts submitted to journal(s)** | 1.3 (3.3)  2/24 | 0.9 (3.9)  6/24 |  |  |  |  |
| Pairwise comparison | Several biomedical journals from a single publisher | **Actual manuscripts submitted to journal(s)** | 1. (3.5)   3/24 | 0.9 (3.6)  6/24 |  |  |  |  |
| Pairwise comparison | Single biomedical journal | **Actual manuscripts submitted to journal(s)** | 0.3 (3.9)  10/24 | -0.3 (3.5)  15/24 |  |  |  |  |
| Pairwise comparison | Several biomedical journals from different publishers | One fabricated manuscript | -0.5 (3.4)  16/24 | -0.1 (3.0)  12/24 |  |  |  |  |
| Pairwise comparison | Several biomedical journals from a single publisher | One fabricated manuscript | -1.2 (3.4)  22/24 | -0.4 (3.9)  16/24 |  |  |  |  |
| Pairwise comparison | Single biomedical journal | One fabricated manuscript | -2.0 (3.1)  24/24 | -0.2 (3.4)  14/24 |  |  |  |  |
| Cluster RCT with randomization of journals | Several biomedical journals from different publishers | **Actual manuscripts submitted to journal(s)** | -0.8 (3.5)  19/24 | -0.4 (3.2)  16/24 | -0.3 (3.9)  6/13 | 1.9 (3.0)  2/10 | -0.8 (3.3)  11/13 | - 1. (3.8)   3/10 |
| Cluster RCT with randomization of journals | Several biomedical journals from a single publisher | **Actual manuscripts submitted to journal(s)** | -0.6 (3.4)  18/24 | -0.7 (3.5)  18/24 | -0.3 (4.2)  6/13 | -0.6 (4.0)  6/10 | -0.6 (3.5)  8/13 | 0.4 (3.7)  5/10 |
| Cluster RCT with randomization of journals | Several biomedical journals from different publishers | One fabricated manuscript | 0.3 (3.5)  10/24 | -2.0 (3.0)  24/24 |  |  |  |  |
| Cluster RCT with randomization of journals | Several biomedical journals from a single publisher | One fabricated manuscript | -0.5 (3.3)  16/24 | -0.1 (3.3)  12/24 |  |  |  |  |
| Interrupted time series analysis | Several biomedical journals from different publishers | **Actual manuscripts submitted to journal(s)** | -1.3 (3.7)  23/24 | -1.2 (3.9)  21/24 | -0.8 (3.4)  11/13 | 0.2 (4.2)  4/10 | -0.4 (3.5)  7/13 | - 1. (3.1)   3/10 |
| Interrupted time series analysis | Several biomedical journals from a single publisher | **Actual manuscripts submitted to journal(s)** | 0.4 (3.4)  8/24 | -1.4 (4.0)  22/24 | -0.3 (3.7)  6/13 | -1.2 (3.5)  9/10 | 0.9 (3.3)  2/13 | -0.1 (3.7)  6/10 |
| Interrupted time series analysis | Single biomedical journal | **Actual manuscripts submitted to journal(s)** | -0.9 (2.8)  20/24 | -0.8 (3.5)  19/24 | -2.8 (2.4)  13/13 | -2.3 (3.6)  10/10 | -1.4 (3.4)  13/13 | -2.1 (2.5)  10/10 |
| Stepped wedge cluster RCT with randomization of journals | Several biomedical journals from different publishers | One fabricated manuscript | -0.3 (4.0)  15/24 | -1.5 (2.9)  23/24 | -0.6 (3.1)  9/13 | -0.6 (3.3)  6/10 | -0.6 (3.0)  8/13 | -0.2 (3.0)  7/10 |
| Stepped wedge cluster RCT with randomization of journals | Several biomedical journals from a single publisher | **Actual manuscripts submitted to journal(s)** | - 1. (3.8)   13/24 | -0.8 (3.5)  19/24 | -0.6 (3.8)  9/13 | -0.3 (3.3)  5/10 | -0.6 (3.3)  8/13 | -1.9 (2.9)  9/10 |

**Appendix 4** – Results – Mean score for each combination of study design features for trust in results

| Study design | | | Interventions | | | | | |
| --- | --- | --- | --- | --- | --- | --- | --- | --- |
| Study Type | Setting | Type of manuscript | TRAINING PEER REVIEWERS  (24 vignettes,  276 pairs)  Mean score (SD)  Rank | RESULTS FREE PEER REVIEW  (24 vignettes, 276 pairs)  Mean score (SD)  Rank | USE OF REPORTING GUIDELINES CHECKLIST  (13 vignettes,  156 pairs*)  Mean score (SD)  Rank | ADDING AN EXPERT TO THE PEER REVIEW PROCESS  (10 vignettes,  90 pairs*)  Mean score (SD)  Rank | USING INCENTIVES  (13 vignettes, 156 pairs*)  Mean score (SD)  Rank | POST- PUBLICATION PEER REVIEW  (10 vignettes, 90 pairs*)  Mean score (SD)  Rank |
| RCT with randomization of manuscripts | Several biomedical journals from different publishers | **Actual manuscripts submitted to journal(s)** | 0.5 (3.3)  7/24 | 1.9 (2.9)  1/24 | 1. **(2.3)**   **1/13** | **2.3 (2.8)**  **1/10** | 0.8 (3.2)  3/13 | **1.6 (3.5)**  **1/10** |
| RCT with randomization of manuscripts | Several biomedical journals from a single publisher | **Actual manuscripts submitted to journal(s)** | 0.6 (3.1)  6/24 | - 1. (3.3)   6/24 | 1.7 (3.0)  3/13 | 1.6 (3.0)  3/10 | 0.7 (3.4)  4/13 | 1.3 (2.9)  2/10 |
| RCT with randomization of manuscripts | Single biomedical journal | **Actual manuscripts submitted to journal(s)** | - 1. (3.5)   14/24 | 1. (3.2)   7/24 | - 1. (3.1)   5/13 | -0.7 (3.8)  8/10 | -0.9 (3.3)  11/13 | -0.9 (3.8)  8/10 |
| RCT with randomization of peer reviewers | Several biomedical journals from different publishers | **Actual manuscripts submitted to journal(s)** | **1.7 (3.0)**  **1/24** | **1.9 (3.1)**  **1/24** | 1.8 (3.2)  2/13 |  | **2.2 (2.8)**  **1/13** |  |
| RCT with randomization of peer reviewers | Several biomedical journals from a single publisher | **Actual manuscripts submitted to journal(s)** | 0.5 (3.0)  7/25 | - 1. (3.2)   4/24 | - 1. (3.4)   4/13 |  | 0.9 (3.2)  2/13 |  |
| RCT with randomization of peer reviewers | Single biomedical journal | **Actual manuscripts submitted to journal(s)** | 0.3 (3.6)  11/24 | 0.4 (3.1)  1/24 | -1.0 (4.2)  12/13 |  | 0.5 (4.1)  6/13 |  |
| RCT with randomization of peer reviewers | Several biomedical journals from different publishers | One fabricated manuscript | 1.0 (2.4)  3/24 | 0.3 (3.2)  4/24 |  |  |  |  |
| RCT with randomization of peer reviewers | Several biomedical journals from a single publisher | One fabricated manuscript | 0.7 (3.8)  5/24 | 0.5 (3.5)  11/24 |  |  |  |  |
| RCT with randomization of peer reviewers | Single biomedical journal | One fabricated manuscript | -1.2 (3.3)  22/24 | 0.1 (3.2)  12/24 |  |  |  |  |
| Pairwise comparison | Several biomedical journals from different publishers | **Actual manuscripts submitted to journal(s)** | - 1. (3.1)   2/24 | 0.7 (2.9)  10/24 |  |  |  |  |
| Pairwise comparison | Several biomedical journals from a single publisher | **Actual manuscripts submitted to journal(s)** | 0.8 (3.5)  4/24 | 0.7 (3.0)  14/24 |  |  |  |  |
| Pairwise comparison | Single biomedical journal | **Actual manuscripts submitted to journal(s)** | - 1. (3.5)   14/24 | -0.4 (3.5)  8/24 |  |  |  |  |
| Pairwise comparison | Several biomedical journals from different publishers | One fabricated manuscript | -0.8 (3.0)  20/24 | 0.1 (3.5)  8/24 |  |  |  |  |
| Pairwise comparison | Several biomedical journals from a single publisher | One fabricated manuscript | -1.6 (2.8)  23/24 | -1.4 (3.4)  17/24 |  |  |  |  |
| Pairwise comparison | Single biomedical journal | One fabricated manuscript | -2.3 (2.8)  24/24 | 0.0 (2.7)  14/24 |  |  |  |  |
| Cluster RCT with randomization of journals | Several biomedical journals from different publishers | **Actual manuscripts submitted to journal(s)** | -0.5 (3.3)  17/24 | 0.2 (3.4)  24/24 | -0.5 (3.9)  8/13 | 1.8 (2.9)  2/10 | -0.8 (3.2)  10/13 | 0.8 (3.4)  4/10 |
| Cluster RCT with randomization of journals | Several biomedical journals from a single publisher | **Actual manuscripts submitted to journal(s)** | -0.2 (3.1)  16/24 | -0.7 (3.1)  16/24 | - 1. (3.7)   5/13 | -0.6 (3.9)  7/10 | -0.6 (3.5)  9/13 | 0.4 (3.5)  5/10 |
| Cluster RCT with randomization of journals | Several biomedical journals from different publishers | One fabricated manuscript | 0.5 (3.3)  7/24 | -2.5(3.1)  13/24 |  |  |  |  |
| Cluster RCT with randomization of journals | Several biomedical journals from a single publisher | One fabricated manuscript | -0.6 (3.0)  18/24 | -0.7 (2.5)  20/24 |  |  |  |  |
| Interrupted time series analysis | Several biomedical journals from different publishers | **Actual manuscripts submitted to journal(s)** | -1.0 (3.6)  21/24 | -1.1 (3.6)  20/24 | -0.8 (3.5)  10/13 | 0.3 (4.2)  4/10 | -1.0 (3.3)  12/13 | - 1. (2.8)   3/10 |
| Interrupted time series analysis | Several biomedical journals from a single publisher | **Actual manuscripts submitted to journal(s)** | - 1. (2.8)   20/24 | -1.3 (3.8)  23/24 | -0.9 (3.6)  11/13 | -1.3 (3.5)  9/10 | 0.7 (3.4)  4/13 | -0.5 (3.7)  7/10 |
| Interrupted time series analysis | Single biomedical journal | **Actual manuscripts submitted to journal(s)** | -0.7 (2.5)  19/24 | -1.1 (3.5)  21/24 | -2.8 (2.5)  13/13 | -2.8 (3.3)  10/10 | -1.5 (3.1)  13/13 | -2.2 (2.4)  10/10 |
| Stepped wedge cluster RCT with randomization of journals | Several biomedical journals from different publishers | One fabricated manuscript | 0.2 (3.4)  12/24 | -0.6 (3.0)  22/24 | -0.2 (2.8)  7/13 | -0.1 (3.3)  5/10 | -0.5 (2.9)  8/13 | - 1. (3.0)   6/10 |
| Stepped wedge cluster RCT with randomization of journals | Several biomedical journals from a single publisher | **Actual manuscripts submitted to journal(s)** | 0.5 (3.2)  7/24 | -0.5 (3.3)  19/24 | -0.5 (3.5)  8/13 | -0.4 (3.4)  6/10 | -0.4 (3.4)  7/13 | -1.9 (2.8)  9/10 |

**Appendix 5** – Results – Mean score for each combination of study design features for feasibility

| Study design | | | Interventions | | | | | |
| --- | --- | --- | --- | --- | --- | --- | --- | --- |
| Study Type | Setting | Type of manuscript | TRAINING PEER REVIEWERS  (24 vignettes,  276 pairs)  Mean score (SD)  Rank | RESULTS FREE PEER REVIEW  (24 vignettes, 276 pairs)  Mean score (SD)  Rank | USE OF REPORTING GUIDELINES CHECKLIST  (13 vignettes,  156 pairs*)  Mean score (SD)  Rank | ADDING AN EXPERT TO THE PEER REVIEW PROCESS  (10 vignettes,  90 pairs*)  Mean score (SD)  Rank | USING INCENTIVES  (13 vignettes, 156 pairs*)  Mean score (SD)  Rank | POST- PUBLICATION PEER REVIEW  (10 vignettes,  90 pairs*)  Mean score (SD)  Rank |
| RCT with randomization of manuscripts | Several biomedical journals from different publishers | **Actual manuscripts submitted to journal(s)** | -0.1 (3.1)  15/24 | -1.0 (2.9)  22/24 | -0.8 (2.9)  11/13 | -1.3 (2.9)  8/10 | -0.8 (3.8)  11/13 | -1.0 (3.8)  7/10 |
| RCT with randomization of manuscripts | Several biomedical journals from a single publisher | **Actual manuscripts submitted to journal(s)** | 1. (2.7)   13/24 | -0.1 (3.3)  16/24 | -0.2 (3.3)  8/13 | 0.3 (3.2)  4/10 | 0.9 (3.4)  3/13 | 0.8 (3.3)  5/10 |
| RCT with randomization of manuscripts | Single biomedical journal | **Actual manuscripts submitted to journal(s)** | 1.3 (2.7)  3/24 | 1.3 (3.1)  2/24 | 1.9 (2.2)  2/13 | 2.3 (2.8)  1/10 | -0.6 (3.3)  9/13 | 1.4 (3.6)  3/10 |
| RCT with randomization of peer reviewers | Several biomedical journals from different publishers | **Actual manuscripts submitted to journal(s)** | -1.3 (3.3)  21/24 | -0.3 (3.8)  17/24 | 0.2 (3.5)  6/13 |  | -0.6 (3.1)  9/13 |  |
| RCT with randomization of peer reviewers | Several biomedical journals from a single publisher | **Actual manuscripts submitted to journal(s)** | -0.3 (3.2)  16/24 | 0.1 (3.0)  12/24 | 0.3 (3.7)  5/13 |  | 1.0 (3.7)  2/13 |  |
| RCT with randomization of peer reviewers | Single biomedical journal | **Actual manuscripts submitted to journal(s)** | 0.5 (3.5)  9/24 | -0.3 (3.6)  17/24 | 1.0 (3.4)  4/13 |  | 0.8 (3.1)  5/13 |  |
| RCT with randomization of peer reviewers | Several biomedical journals from different publishers | One fabricated manuscript | 0.7 (3.0)  8/24 | 0.7 (2.9)  4/24 |  |  |  |  |
| RCT with randomization of peer reviewers | Several biomedical journals from a single publisher | One fabricated manuscript | 0.2 (3.5)  11/24 | 0.3 (2.8)  9/24 |  |  |  |  |
| RCT with randomization of peer reviewers | Single biomedical journal | One fabricated manuscript | 1.2 (3.2)  6/24 | 0.4 (3.2)  8/24 |  |  |  |  |
| Pairwise comparison | Several biomedical journals from different publishers | **Actual manuscripts submitted to journal(s)** | 0.9 (3.0)  7/24 | 0.0 (3.3)  14/24 |  |  |  |  |
| Pairwise comparison | Several biomedical journals from a single publisher | **Actual manuscripts submitted to journal(s)** | 0.4 (3.1)  10/24 | 0.5 (3.6)  6/24 |  |  |  |  |
| Pairwise comparison | Single biomedical journal | **Actual manuscripts submitted to journal(s)** | 2.4 (2.8)  2/24 | 0.5 (3.7)  6/24 |  |  |  |  |
| Pairwise comparison | Several biomedical journals from different publishers | One fabricated manuscript | 1.0 (3.1)  6/24 | -0.7 (3.2)  21/24 |  |  |  |  |
| Pairwise comparison | Several biomedical journals from a single publisher | One fabricated manuscript | 1.2 (3.1)  4/24 | 0.2 (3.4)  11/24 |  |  |  |  |
| Pairwise comparison | Single biomedical journal | One fabricated manuscript | **2.6 (2.4)**  **1/24** | **1.7 (2.9)**  **1/24** |  |  |  |  |
| Cluster RCT with randomization of journals | Several biomedical journals from different publishers | **Actual manuscripts submitted to journal(s)** | -0.9 (3.4)  19/24 | 0.0 (3.2)  14/24 | -0.5 (3.6)  9/13 | 0.3 (2.8)  4/10 | 0.5 (3.2)  6/13 | -0.5 (3.4)  6/10 |
| Cluster RCT with randomization of journals | Several biomedical journals from a single publisher | **Actual manuscripts submitted to journal(s)** | -0.4 (3.3)  17/24 | 0.3 (3.5)  9/24 | **2.0 (3.1)**  **1/13** | -0.7 (3.1)  7/10 | 0.4 (3.3)  8/13 | 1.1 (3.2)  4/10 |
| Cluster RCT with randomization of journals | Several biomedical journals from different publishers | One fabricated manuscript | -0.7 (3.2)  18/24 | -0.3 (3.2)  17/24 |  |  |  |  |
| Cluster RCT with randomization of journals | Several biomedical journals from a single publisher | One fabricated manuscript | 0.2 (3.2)  11/24 | 0.7 (2.6)  4/24 |  |  |  |  |
| Interrupted time series analysis | Several biomedical journals from different publishers | **Actual manuscripts submitted to journal(s)** | -1.4 (2.9)  22/24 | -0.5 (3.6)  20/24 | -0.5 (3.6)  9/13 | 0.3 (3.8)  4/10 | 0.5 (3.2)  6/13 | -1.3 (2.7)  8/10 |
| Interrupted time series analysis | Several biomedical journals from a single publisher | **Actual manuscripts submitted to journal(s)** | -0.9 (3.2)  19/24 | 0.1 (4.0)  12/24 | 0.0 (3.2)  7/13 | 0.7 (3.7)  3/10 | 0.9 (3.5)  3/13 | 1.5 (3.2)  2/10 |
| Interrupted time series analysis | Single biomedical journal | **Actual manuscripts submitted to journal(s)** | 0.0 (3.3)  13/24 | 1.0 (3.1)  3/24 | 1.5 (3.4)  3/13 | **2.3 (2.0)**  **1/10** | **1.2 (3.8)**  **1/13** | **2.3 (2.1)**  **1/20** |
| Stepped wedge cluster RCT with randomization of journals | Several biomedical journals from different publishers | One fabricated manuscript | -3.7 (2.1)  24/24 | -2.1 (3.2)  23/24 | -2.5 (1.8)  12/13 | -2.2 (2.5)  10/10 | -2.8 (2.0)  13/13 | -2.8 (1.4)  10/10 |
| Stepped wedge cluster RCT with randomization of journals | Several biomedical journals from a single publisher | **Actual manuscripts submitted to journal(s)** | -2.8 (2.9)  23/24 | -2.7 (3.4)  24/24 | -2.5 (3.2)  12/13 | -1.9 (2.3)  9/10 | -1.3 (3.2)  12/13 | -1.5 (3.1)  9/10 |

**Appendix 6**– Results – Features associated with trust in the results: parameter estimates [and 95% confidence intervals]. For each independent variable, parameter estimates represent mean difference in trust in the results associated with each category of independent variable as compared with the reference (after adjusting for all other variables in the table).

| VARIABLE** | Intervention | | | | | |
| --- | --- | --- | --- | --- | --- | --- |
|  | **TRAINING PEER REVIEWERS**  **(24 vignettes,  276 pairs)**  **Estimate [95% CI]** | **RESULTS FREE PEER REVIEW**  **(24 vignettes,  276 pairs)**  **Estimate [95% CI]** | **USE OF REPORTING GUIDELINES CHECKLIST**  **(13 vignettes,  156 pairs*)**  **Estimate [95% CI]** | **ADDING AN EXPERT TO THE PEER REVIEW PROCESS**  **(10 vignettes,  90 pairs*)**  **Estimate [95% CI]** | **USING INCENTIVES**  **(13 vignettes,  156 pairs*)**  **Estimate [95% CI]** | **POST-PUBLICATION PEER REVIEW**  **(10 vignettes,  90 pairs*)**  **Estimate [95% CI]** |
| Study type | | | | | | |
| RCT with randomization of manuscripts | 0.37  [-0.88 ; 1.57] | **2.03**  **[0.82 ; 3.18]** | **2.31**  **[1.11 ; 3.46]** | **2.02**  **[0.71 ; 3.51]** | 1.07  [-0.23 ; 2.34] | **2.27**  **[0.89 ; 3.79]** |
| RCT with randomization of peer reviewers | **0.95**  **[-0.26 ; 2.12]** | 1.89  [0.75 ; 3.03] | 1.76  [0.50 ; 3.10] |  | **2.04**  **[0.71 ; 3.29]** |  |
| Cluster RCT with randomization of journals | -0.02  [-1.26 ; 1.24] | 0.14  [-0.99 ; 1.28] | 0.16  [-1.37 ; 1.78] | 0.61  [-1.00; 2.27] | -0.28  [-1.63 ; 1.07] | 1.33  [-0.17 ; 3.00] |
| Interrupted time series analysis | -0.51  [-1.69 ; 0.83] | -0.54  [-1.80 ; 0.86] | -0.40  [-1.60 ; 0.82] | -0.42  [-2.01 ; 1.19] | 0.27  [-0.95 ; 1.54] | 1.10  [-0.16 ; 2.58] |
| Pairwise comparison | 0.03  [-0.26 ; 1.23] | 1.08  [-0.04 ; 2.28] |  |  |  |  |
| Stepped wedge cluster RCT with randomization of journals*** | 0.00  [-] | 0.00  [-] | 0.00  [-] | 0.00  [-] | 0.00  [-] | 0.00  [-] |
| Setting | | | | | | |
| Single biomedical journal | -1.01  [-1.77 ; -0.27] | -0.48  [-1.22 ; 0.35] | -2.30  [-3.34 ; -1.27] | -2.85  [-4.48 ; -1.07] | -1.31  [-2.41 ; -0.25] | -3.11  [-4.57 ; -1.57] |
| Several biomedical journals from a single publisher | -0.20  [-0.79 ; 0.40] | 0.17  [-0.80 ; 0.42] | -0.19  [-1.00 ; 0.67] | -1.19  [-2.39 ; -0.04] | **0.12**  **[-0.69 ; 0.92]** | -1.21  [-2.15 ; 0.21] |
| Several biomedical journals from different publishers*** | **0.00**  **[-]** | **0.00**  **[-]** | **0.00**  **[-]** | **0.00**  **[-]** | 0.00  [-] | **0.00**  **[-]** |
| Type of manuscript | | | | | | |
| Actual manuscripts submitted to journal(s) | **1.05**  **[0.37 ; 1.70]** | **0.96**  **[0.31 ; 1.54]** |  |  |  |  |
| One fabricated manuscript*** | 0.00  [-] | 0.00  [-] |  |  |  |  |

*Indicates the pairs of vignettes for these interventions were assessed twice each

**Estimation of fixed effect takes into account the reading order of the 2 vignettes of a pair

***Indicates the reference category for each independent variable

Diagonal line in the table cell represents non-applicable data.

**Appendix 7** – Results – Feature associated with feasibility: parameter estimates [and 95% confidence intervals]. For each independent variable, parameter estimates represent mean difference in feasibility associated with each category of independent variable as compared with the reference (after adjusting for all other variables in the table).

| VARIABLE** | Intervention | | | | | |
| --- | --- | --- | --- | --- | --- | --- |
|  | **TRAINING PEER REVIEWERS**  **(24 vignettes,  276 pairs)**  **Estimate [95% CI]** | **RESULTS FREE PEER REVIEW**  **(24 vignettes, 276 pairs)**  **Estimate [95% CI]** | **USE OF REPORTING GUIDELINES CHECKLIST**  **(13 vignettes,  156 pairs*2)**  **Estimate [95% CI]** | **ADDING AN EXPERT TO THE PEER REVIEW PROCESS**  **(10 vignettes,  90 pairs*2)**  **Estimate [95% CI]** | **USING INCENTIVES**  **(13 vignettes,  156 pairs*2)**  **Estimate [95% CI]** | **POST-PUBLICATION PEER REVIEW**  **(10 vignettes,  90 pairs*2)**  **Estimate [95% CI]** |
| Study type | | | | | | |
| RCT with randomization of manuscripts | 3.23  [2.22 ; 4.21] | 2.17  [0.84 ; 3.36] | 2.19  [1.07 ; 3.29] | 1.84  [0.75 ; 2.99] | 1.89  [0.75 ; 3.03] | 2.01  [0.74 ; 3.20] |
| RCT with randomization of peer reviewers | 2.71  [1.72 ; 3.01] | 2.12  [0.93 ; 3.24] | 2.49  [1.35 ; 3.60] |  | 2.46  [1.25 ; 3.68] |  |
| Cluster RCT with randomization of journals | 2.44  [1.40 ; 3.52] | **2.38**  **[1.16 ; 3.51]** | **3.22**  **[2.01 ; 4.37]** | 2.09  [0.83 ; 3.41] | 2.56  [1.27 ; 3.71] | 2.23  [0.97 ; 3.44] |
| Interrupted time series analysis | 1.99  [0.86 ; 3.16] | 2.32  [0.96 ; 3.57] | 2.27  [1.23 ; 3.31] | **2.60**  **[1.34 ; 3.82]** | **2.89**  **[1.68 ; 4.01]** | **2.32**  **[1.12 ; 3.52]** |
| Pairwise comparison | **3.89**  **[2.91 ; 4.89]** | 2.35  [1.05 ; 3.58] |  |  |  |  |
| Stepped wedge cluster RCT with randomization of journals*** | 0.00  [-] | 0.00  [-] | 0.00  [-] | 0.00  [-] | 0.00  [-] | 0.00  [-] |
| Setting | | | | | | |
| Single biomedical journal | **1.44**  **[0.77; 2.13]** | **1.01**  **[0.25 ; 1.78]** | **1.83**  **[0.88 ; 2.82]** | **2.27**  **[0.95 ; 3.62]** | 0.64  [-0.41 ; 1.70] | **2.34**  **[0.91 ; 3.72]** |
| Several biomedical journals from a single publisher | 0.37  [-0.24 ; 0.98] | 0.38  [-0.21 ; 1.04] | 0.65  [-0.20 ; 1.47] | 0.25  [-0.73 ; 1.32] | **1.02**  **[0.15 ; 1.91]** | 1.47  [0.40 ; 2.43] |
| Several biomedical journals from different publishers*** | 0.00  [-] | 0.00  [-] | 0.00  [-] | 0.00  [-] | 0.00  [-] | 0.00  [-] |
| Type of manuscript | | | | | | |
| Actual manuscripts submitted to journal(s) | -0.65  [-1.32 ; 0.03] | -0.27  [-0.96 ; 0.36] |  |  |  |  |
| One fabricated manuscript*** | **0.00**  **[-]** | **0.00**  **[-]** |  |  |  |  |

*Indicates the pairs of vignettes for these interventions were assessed twice each

**Estimation of fixed effect takes into account the reading order of the 2 vignettes of a pair

***Indicates the reference category for each independent variable

Diagonal line in the table cell represents non-applicable data.
